# Impact of COVID-19 on psychosocial work factors and emotional exhaustion among healthcare workers

**DOI:** 10.1093/eurpub/ckac130.130

**Published:** 2022-10-25

**Authors:** F van Elk, SJW Robroek, A Burdorf, KM Oude Hengel

**Affiliations:** 1 Public Health, Erasmus University Medical Center, Rotterdam, Netherlands; 2 Work Health Technology, TNO, Leiden, Netherlands

## Abstract

**Background:**

Healthcare workers are at risk to develop mental health problems and to experience adverse psychosocial working conditions during the COVID-19 pandemic. This study aims to investigate across subgroups of healthcare workers i) differences in psychosocial working conditions and emotional exhaustion, ii) changes in psychosocial working conditions and emotional exhaustion during the pandemic compared to the situation before, and iii) impact of different stages of the COVID-19 pandemic in terms of hospital pressure on psychosocial working conditions and emotional exhaustion.

**Methods:**

Data on psychosocial working conditions and emotional exhaustion of five measurements from 1,915 healthcare workers participating in the longitudinal study ‘the Netherlands Working Conditions Survey- COVID-19’ were used. Three subgroups were defined: working with COVID-19 patients, working with other patients, and not working with patients. For each measurement, hospital pressure was determined. Linear mixed models were fitted to analyze the differences across subgroups of healthcare workers.

**Results:**

Healthcare workers working with patients, in particular COVID-19 patients, had more unfavorable psychosocial working conditions than those not working with patients. Psychosocial working conditions deteriorated among those working with patients compared to pre-COVID-19, but no changes were found for emotional exhaustion. An increasing hospital pressure resulted in improved job autonomy and emotional demands among healthcare workers working with COVID-19 patients, but did not result in differences in other working conditions and emotional exhaustion.

**Conclusions:**

Psychosocial working conditions deteriorated for healthcare workers working with (COVID-19) patients during the pandemic compared to the situation before the pandemic, while emotional exhaustion did not change in these subgroups. This shows the importance of interventions to improve working conditions of healthcare workers.

**Key messages:**

- Healthcare workers working with (COVID-19) patients experience unfavorable psychosocial working conditions, which deteriorated during COVID-19. No changes were found for emotional exhaustion.
- An increase in hospital pressure did not further deteriorate psychosocial working conditions.

